# Characterization of Chitosan Extracted from Fish Scales of the Colombian Endemic Species *Prochilodus magdalenae* as a Novel Source for Antibacterial Starch-Based Films

**DOI:** 10.3390/polym13132079

**Published:** 2021-06-24

**Authors:** Carlos Molina-Ramírez, Paulina Mazo, Robin Zuluaga, Piedad Gañán, Juan Álvarez-Caballero

**Affiliations:** 1 Grupo Química y Bioprospección de Productos Naturales, Universidad del Magdalena, Santa Marta 470004, Colombia; jalvarez@unimagdalena.edu.co; 2 Grupo de Investigación Sobre Nuevos Materiales (GINUMA), Universidad Pontificia Bolivariana, Medellín 050004, Colombia; piedad.ganan@upb.edu.co; 3 Grupo de Investigaciones Agroindustriales (GRAIN), Universidad Pontificia Bolivariana, Medellín 050004, Colombia; paulina.mazo@upb.edu.co (P.M.); robin.zuluaga@upb.edu.co (R.Z.)

**Keywords:** chitosan, fish scales, *Prochilodus magdalenae*, antibacterial agent

## Abstract

Scales of *Prochilodus magdalenae*, a Colombian endemic fish species, were used to obtain chitosan for application as an antibacterial agent integrated into starch-based films. Analysis of its composition during the demineralization and deproteinization process indicated that minerals and protein were both removed successfully. At this point, mild conditions for the deacetylation process were employed, namely, 2, 4, and 6 wt.% NaOH at room temperature for 16 h. Chitosan processed under 2 wt.% NaOH had low molecular weight, with the lowest value of 107.18 ± 24.99 kDa, which was closely related to its antibacterial activity. Finally, this chitosan was integrated into a banana starch-based film, and its antibacterial activity was assayed in *Escherichia coli* and *Staphylococcus aureus* cultures, with positive results in the former culture, especially due to the low-molecular-weight characteristic of chitosan.

## 1. Introduction

Chitosan is a derivative of chitin, which is one of the three most important and abundant polysaccharides on the planet, together with cellulose and starch [1,2]. Chitin is a structural polysaccharide similar to white, inelastic, and nitrogenous cellulose consisting of 2-acetamido-2 deoxy β-D-glucose linked together by β-1,4 linkages conventionally extracted from insects, fungi, annelids, mollusks, and crustaceans, although industrial production occurs particularly from shrimp and crab crustaceans [2,3,4].

**Figure 1:**
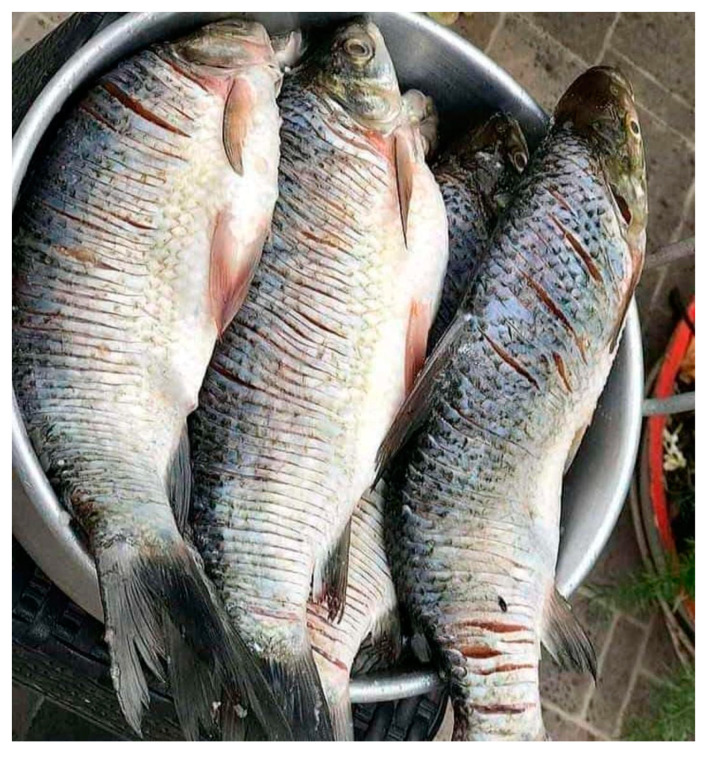
“Bocachico” commercialized informally in the municipality of Plato, Magdalena, Colombia.

As mentioned above, chitosan can be obtained from different natural sources, including fish scales, which are composed mainly of collagen and carbonates/hydroxyapatite [5], and minimally explored components such as chitin, which can be converted into chitosan [6]. Fish scales can be used in many applications such as filling material for paper [7], biomass for energy generation [5], or removing heavy metals [8]. However, fish scales are discarded because they are considered waste produced on a large scale, constituting 1 wt.% of whole weight in fish, which makes it one of the major sources of contamination in riverine regions in many parts of the world [9]. *P. magdalenae*, commonly called “bocachico” in Colombia, is an endemic species grown naturally mainly in the Magdalena River and Cauca River and can be used as a source to obtain scales to extract chitosan. “Bocachico” is highly commercialized informally by street vendors, as exhibited in Figure 1, because it constitutes an important food source for riverine populations [10,11]. Likewise, the importance of using local sources to obtain chitosan is due to the significant effect on its properties such as solubility, reactivity, affinity for solvents, and swelling [3].

Chitosan is a nontoxic, biocompatible, biodegradable and polyelectrolyte material that inhibits the growth of a wide variety of fungi, bacteria, and yeasts, which has allowed it to have applications in different areas such as cosmetics, agriculture, food, biomedicals, textiles [12,13]. It also has been used for the removal of emerging pollutants such as antibiotics [14] and as a flocculant for treatment of wastewater [15]. Additionally, it is considered generally recognized as safe (GRAS) [16], which has allowed applications through combination with other polymers such as starch for the development of biodegradable packaging for food [13]. The antimicrobial activity of chitosan depends on various conditions such as the pH, type of microorganism, pKa, and degree of deacetylation, among others [13]; however, chitosan is an alternative as a natural preservative against food spoilation by microorganisms [17], replacing conventional preservatives such as potassium sorbate or sodium benzoate [18].

Therefore, this paper proposes a protocol to extract chitosan from highly polluting riverbanks, such as that from the scales of *Prochilodus magdalenae*, an endemic species in Colombia, as a novel source for this material and to characterize its use as a possible antimicrobial agent integrated into starch-based films, with a high potential to produce biodegradable food packaging.

## 2. Materials and Methods

### 2.1. Feedstock and Reagents

*P. magdalenae* scales were collected in the fish market square of Plato, Magdalena-Colombia, after the flaking process by artisanal fishermen of the city. Then, the scales were washed with plenty of tap water to clean organic debris and were stored at −20 °C until reaching the required quantity. Once the collection was completed, the scales were sent to Medellin, Colombia, to be processed, and chitosan was obtained.

Low-molecular-weight chitosan was purchased from Sigma-Aldrich (Sigma-Aldrich Co., Milwaukee, WI, USA) for comparison to chitosan obtained in this research, and sodium hydroxide and hydrochloric acid were purchased from Merck (Merck KGaA, Darmstadt, Germany). All the reactives used in this research were reagent grade.

### 2.2. Sample Preparation

The scales were dried as a previous step for chitosan extraction. The drying process was carried out in an oven at 60 °C until a constant weight was reached. Then, the scale size was reduced using a blender at 3000 rpm for 3 min.

### 2.3. Chitosan Extraction

**Figure 2:**
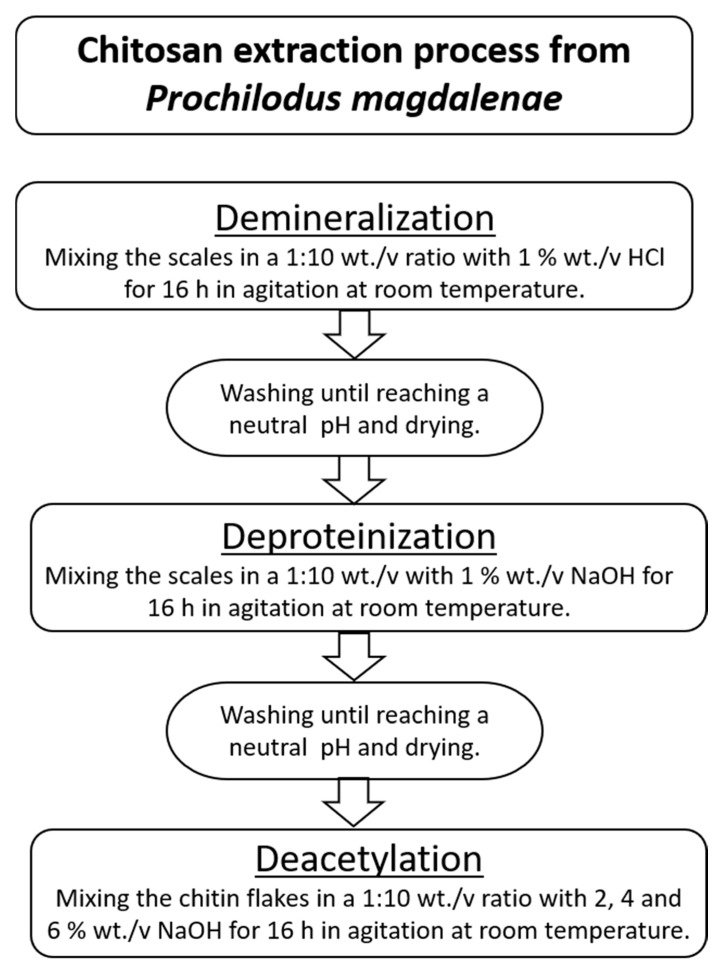
Chitosan extraction process from *P. magdalenae* scales.

Chitosan extraction involved three main stages: demineralization, deproteinization, and deacetylation. In Figure 2, the process to obtain chitosan from *P. magdalenae* scales is described. Deacetylation was performed with sodium hydroxide (NaOH) at a concentration of 50% wt./v and a temperature in the range of 60–90 °C.

### 2.4. Chitosan Characterization

The properties and characteristics of the chitosan samples obtained after deacetylation at different NaOH concentrations were analyzed using the following techniques. Before analysis, the samples were dried in an oven at 50 °C until a reaching constant weight, milled, and sieved through 100 mesh.

#### 2.4.1. Protein Determination

To quantify protein removal after chemical treatment with NaOH, Biuret’s method, modified and adapted by Gornall et al. (1949), was used [19]. All samples (0.1 g) were weighed and dispersed in 100 mL of distilled water and then vortexed and sonicated for 10 min to facilitate protein solubilization. Next, the samples were centrifuged at 6000 rpm, and 1 mL of supernatant was withdrawn from the samples to quantify the protein concentration. Finally, protein calculations were performed based on the dry weight of the samples. All analyses were performed in triplicate.

#### 2.4.2. Mineral Quantification (Phosphorous and Calcium)

The mineral content in the *P. magdalenae* scales and chitin was determined using an ICE^®^ 3500 atomic absorption spectrometer according to EPA method EPA-3050-B for digestion of the sample and Standard Method SM-3111-D to calculate the Ca content by atomic absorption spectroscopy (AAS). For phosphorous determination, the standard methods, SM-4500-P.B and SM-4500-P.E, were employed for acid digestion and P quantification by the spectrophotometric method, respectively. All values have a deviation of less than 5%.

#### 2.4.3. Attenuated Total Reflection Fourier Transform Infrared (FTIR–ATR) Spectroscopy

The spectra were obtained on a Nicolet iS50 spectrophotometer (Thermo Scientific, Waltham, MA, USA) in the range of 40,000–400 cm^−1^ using a diamond ATR crystal. The spectra were recorded with a resolution of 4 cm^−1^ and an accumulation of 64 scans.

ATR correction was performed by Omnic software (Thermo Fisher Scientific, Waltham, MA, USA), and the baseline was defined manually.

The samples were dried at 50 °C for 2 d before the assay.

#### 2.4.4. Scanning Electron Microscopy (SEM)

Chitosan samples were coated with gold/palladium using ion sputtering. The samples were observed with a JEOL^®^ JSM 5910 LV microscope (JEOL Ltd., Tokyo, Japan) operated at 15 kV under high vacuum.

The samples were dried at 50 °C for 2 d and milled before the assay.

#### 2.4.5. Thermogravimetric Analysis (TGA)

The thermal degradation of the samples was evaluated in a Mettler Toledo TGA/SDTA 851E thermogravimetric analyzer (Mettler Toledo, Columbus, OH, USA). Dried and ground samples were weighed and heated in a nitrogen atmosphere from room temperature to 800 °C at a heating rate of 10 °C min^−1^.

#### 2.4.6. Differential Scanning Calorimetry (DSC)

A DSC TA Q2000 Series system (TA Instruments, New Castle, DE, USA) was employed to carry out the differential scanning calorimetry analyses. Five milligrams of sample were placed into aluminum sealed pans, and scans were made from 0 °C to 250 °C at a 10 °C/min rate in a nitrogen atmosphere.

#### 2.4.7. Viscosity Molecular Weight

The viscosity average molecular weight (MW) was determined by using a #75 Ostwald viscometer operated at a temperature of 25 °C. Chitosan was dissolved in 0.30 M acetic acid/0.2 M sodium acetate buffer, pH 4.5. Intrinsic viscosities were determined by extrapolation of the average values of flow time obtained for five solutions of different concentrations. The viscosity average molecular weight is calculated according to the Mark–Houwink–Sakurada equation (Equation (1)).

(1) $$[\eta ]=K{\left({\bar{M}}_{v}\right)}^{a}$$

where [*η*] is the intrinsic viscosity, ${\bar{M}}_{v}$ is the viscosity average molecular weight, and *K* and *a* are constant parameters equivalent to 74 × 10^−3^ mL/g and 0.76, respectively [20].

#### 2.4.8. Deacetylation Degree

For the deacetylation degree, 0.1 g of sample was added to 10 mL of 0.2 M standard HCl solution. Then, the solutions were brought to 100 mL with distilled water and 0.746 g of KCl to adjust the ionic strength to 0.1. The titration processes were carried out with 0.1 M NaOH solution. A pH meter (SI Analytics LAB 850, Xylem Analytics, Weilheim in Oberbayern, Germany) was used for pH measurements under stirring conditions. The assays started at approximately pH 2 and went to pH 11. The method to determine the deacetylation degree (*DD* %) was the potentiometric titration method, and Equation (2) was used.

(2) $$DD(\%)=2.03\frac{V_{2}-V_{1}}{m+0.0042(V_{2}-V_{1})}$$

where *m* is the weight of the sample; *V*_1_ and *V*_2_ are the volume of NaOH solution at the inflection points; 2.03 is the coefficient correlated to the molecular weight of the monomer of chitin; and 0.0042 is the difference between monomers of chitin and chitosan [21].

#### 2.4.9. Evaluation of Antibacterial Effect in the Starch-Based Film

To assess the antibacterial activity of chitosan in food packaging, a banana starch-based film was prepared according to a slightly modified method developed by Leites et al. (2018) [22]. Before film manufacturing, a 4.5 wt.% chitosan solution was prepared in 2 v. % acetic acid. The film was obtained by casting after adding chitosan (20 wt.%) into the mix of starch (4 wt.%), glycerol (1.5 wt.%), and water (74.5 wt.%). Once the film was dried, a sample of 1 cm diameter was taken and placed on separate *Escherichia coli* and *Staphylococcus aureus* cultures to evaluate the effect of chitosan as an antibacterial agent. The culture was grown for 3 d, and the effect of chitosan was measured by the formation of an inhibition halo around the film sample.

## 3. Results and Discussion

The extraction of chitosan from *P. magdalenae* was performed under the conditions shown in Figure 2. First, 45.2% chitin was recovered, which was slightly higher than the amount obtained by Lavall et al. (2007), who achieved a 40–42% recovery [23]. After the demineralization and deproteinization treatments, some chemical changes occurred on the scales, as shown in Table 1. These results show that a major quantity of non-chitosan components was removed from the scales; in the case of minerals, 99.99% of phosphorous and 98.51% of calcium were removed, and for protein, the removal yield was approximately 75.91%, which must be eliminated before to facilitate the deacetylation process. Then, the next step was deacetylation under three different concentrations of NaOH, namely, 2% (Chi-2), 4% (Chi-4), and 6% (Chi-6), to determine their effect on the chitosan properties. In this regard, in previous experiments, information was collected that pointed out the reason for using mild conditions during the chitin deacetylation process in this research: when 50 wt.% NaOH and 80–90 °C conditions were applied, the isolated chitin was degraded because it was β-chitin (data not shown). The above phenomenon was observed by Lavall et al. (2007), who obtained β-chitin samples that were more deacetylated due to the high susceptibility of this polymorph to the treatments employed during chitosan extraction from squid pens [23].

To determine which treatment is more convenient to obtain chitosan from *P. magdalenae* scales for use as an antimicrobial agent, the first properties evaluated were the deacetylation degree (*DD*) and molecular weight (MW), which are reported in Table 2. Regarding this topic, significant differences were not found in the MW of chitosan obtained by changing the NaOH concentration, which could be related to the high reactivity of β-chitin [24]. According to the classification of chitosan, it can exist in three categories: low-molecular-weight (less than 150 kDa), medium-molecular-weight (from 150 kDa to 700 kDa), and high-molecular-weight (higher than 700 kDa) chitosan [25]. In Table 2, it can be observed that the MW in the three samples obtained is consistent with low-molecular-weight chitosan. This type of chitosan is very useful as an antimicrobial agent due to its interaction with phospholipids in membranes by a chelation mechanism [26,27]. In this research, the sample Chi-2% was selected for use as the antimicrobial component in a starch-based film because it implies a lower quantity of NaOH used.

**Figure 3:**
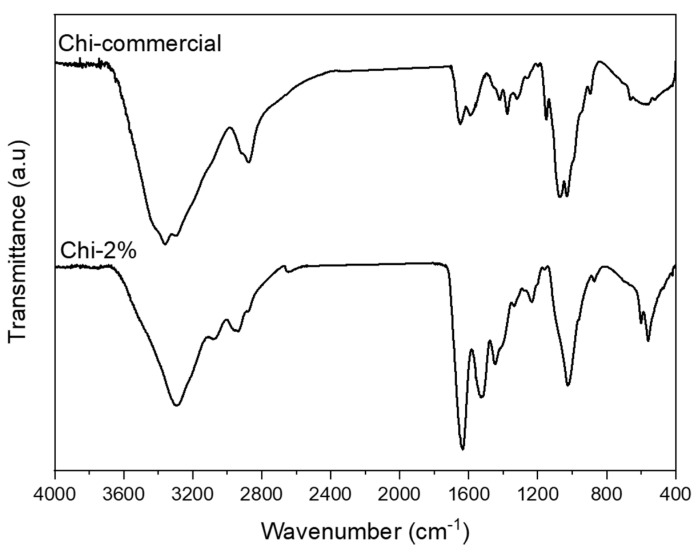
FTIR–ATR spectra recorded for Chi-commercial and chitosan (Chi-2%) extracted from *P. magdalenae* scales.

Once the 2 wt.% NaOH treatment was selected, FTIR–ATR spectra were recorded for Chi-2% and Chi-commercial to study their chemical composition, as shown in Figure 3. The FTIR spectra of Chi-2% and Chi-commercial are shown in Figure 3. In the spectra, the peak at 564 cm^−1^ was assigned to NH out-of-plane bending in amide groups, and the 1160 cm^−1^ peak was assigned to C–O–C stretching [28]. Shown also is a 1026 cm^−1^ band corresponding to the free amino group of glucosamine at C_2_ 871 cm^−1^ related to C–N stretching, a 1545 cm^−1^ band assigned to the coupling of N–H bending and C–N stretching in amide II [6,23,29] and a band at 1640 cm^−1^ corresponding to amide I [30]. Likewise, the spectrum of Chi-2% shows the presence of certain bands such as that at 3073 cm^−1^, which is assigned to amide B [31] related to proteins remaining in the sample, as shown in Table 1, and, unlike Kumari et al. (2017), they remain as β-chitosan because bands at 1604 or 1423 cm^−1^ were not formed [32]. In addition, a 3360 cm^−1^ band is observed in the Chi-commercial spectrum, which is not observed in Chi-2%; this finding is related to the lower intra- and inter-molecular hydrogen bonding characteristics of β-chitosan derived from β-chitin [33].

**Figure 4:**
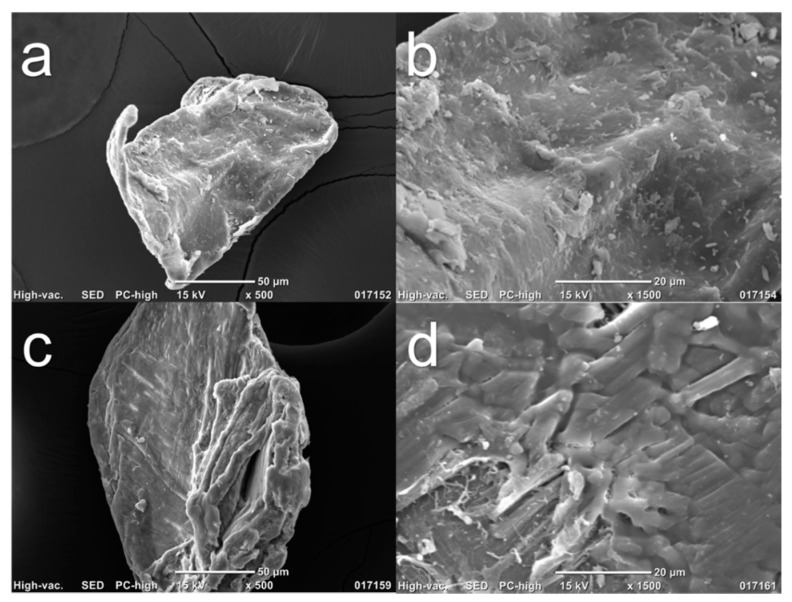
SEM images of chitosan flakes (**a**) and (**b**) are Chi-commercial (control) images, and (**c**) and (**d**) are Chi-2% images.

After evaluation using ATR–FTIR, SEM images of Chi-commercial and Chi-2% were captured to compare their surface characteristics and the effect of the 2% NaOH treatment, as shown in Figure 4. Figure 4a,c shows the morphology of the Chi-commercial and Chi-2% chitosan flakes, respectively, and both showed an irregular shape, as was reported by Kim et al. [34]. In Figure 4b,d, a magnified image is reported. These results demonstrate the fibrillar nature of chitosan, especially in Chi-2%, as was found by Kumari and Rath in 2014 [6]. Likewise, Chi-2% exhibits a cracked surface compared to the smoother surface of Chi-commercial, which is because β-chitin is more sensitive to the chemical treatments used, as mentioned above.

**Figure 5:**
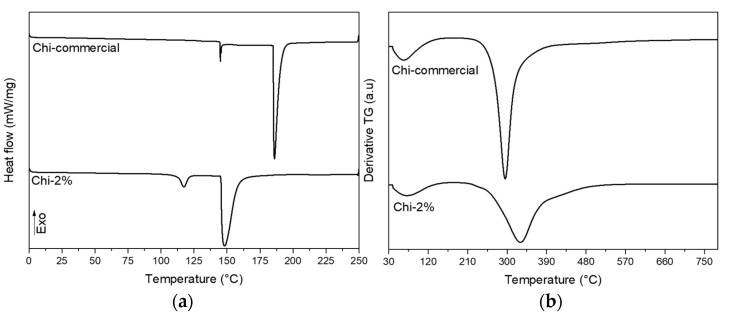
(**a**) DSC and (**b**) DTG thermograms of commercial chitosan and chitosan obtained from *P. magdalenae* scales.

Once the surface and chemical characteristics of the chitosan obtained by the mild condition treatment of 2% NaOH were determined, thermal analyses with DTG and DSC were carried out, and the results are shown in Figure 5a,b. The results showed that the sample of chitosan extracted from *P. magdalenae* scales was very similar to commercial low-molecular-weight chitosan. Thermal analysis showed a sharp peak for Chi-commercial in Figure 5, which indicates that this sample is purer than Chi-2%. In addition, DTG of Chi-commercial presents major degradation at approximately 295 °C, unlike Chi-2%, which shows a broad peak of maximum degradation at approximately 330 °C, reported to be from the thermal rupture of glycosidic linkages in pyranose rings [35]. This result suggests a variable molecular weight in Chi-2%, which is correlated to the three different endothermic events presented in the DSC thermogram. Table 3 shows the value of the main thermal transformation extracted from the DSC thermogram. It is important to highlight that for polymers, the melting phenomenon (peak temperature) is an average of the melting temperature of crystallites, which is not a characteristic value for the sample but rather, depends on the MW [36,37]. Therefore, in Table 3, it can be appreciated that both samples evaluated present different values for onset temperature. Likewise, chitosan needs high thermal energy to initiate its thermal decomposition [38], and for the samples evaluated, a value of 195.66 J/g dry weight and 143.23 J/g dry weight was determined for Chi-commercial and Chi-2%, respectively, which is related to the MW previously calculated for each sample.

**Figure 6:**
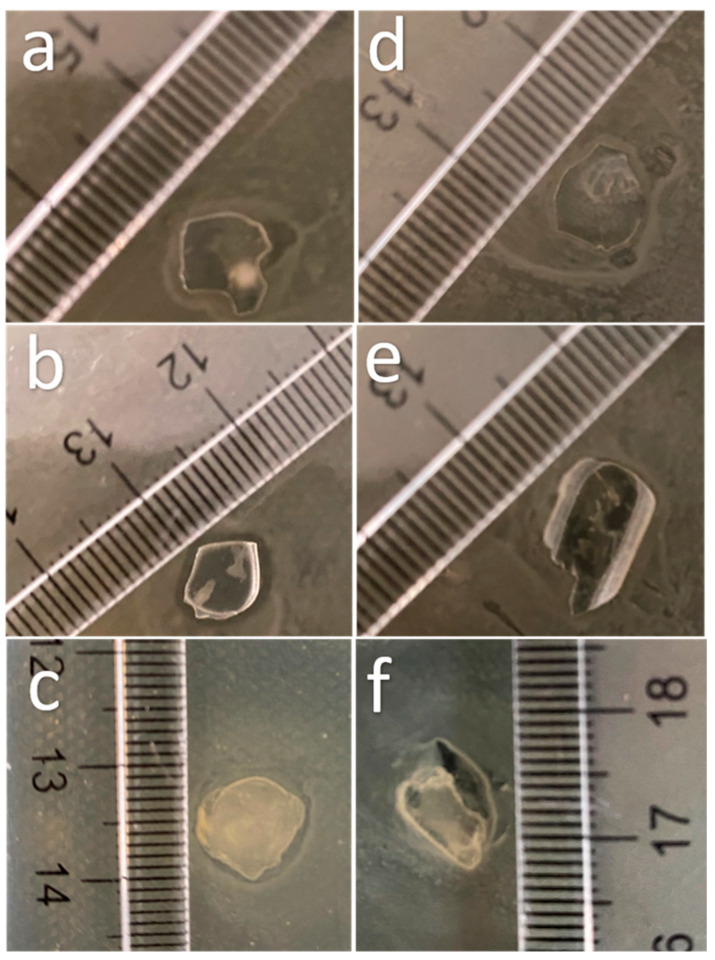
Images of the effect of starch-based films reinforced with chitosan extracted from *P. magdalenae* scales on *E. coli* culture (**a**) without chitosan, (**b**) with Chi-commercial and (**c**) with Chi-2%; and on *S. aureus* culture (**d**) without chitosan, (**e**) with Chi-commercial and (**f**) with Chi-2%.

Finally, the Chi-2% sample was assayed in vitro to determine its antimicrobial effect against *Escherichia coli* (Gram-negative) and *Staphylococcus aureus* (Gram-positive), and the results are shown in Figure 6. Figure 6a,d shows the response of bacteria to the control film (without chitosan), which had no effect on the cultures. Figure 6b shows an inhibition halo of approximately 1 mm around the starch-based film with Chi-commercial added; however, this did not happen with *S. aureus* (Figure 6e). Finally, Figure 6c,f shows the effect of the starch-based film containing chitosan obtained in this research (Chi-2%), with an inhibition halo around the film in *E. coli* culture similar to that with Chi-commercial. As discussed above, low-molecular-weight (low MW) chitosan can enhance the antimicrobial effect; however, according to Zheng and Zhu (2003), this does not apply to all microorganisms; i.e., in their research, they found that high MW improved the inhibitory effect on *S. aureus* because, in this way, it inhibited nutrient absorption, but for *E. coli,* low MW chitosan was more efficient than high MW chitosan in this application because lower MW chitosan can penetrate microbial cells more easily and disrupt cell metabolism [39].

## 4. Conclusions

*Prochilodus magdalenae* scales, a contaminant of riparian zones of rivers in Colombia, were proven to be a novel feedstock to obtain chitosan such as commercial chitosan of low molecular weight, which reduced Gram-negative microorganism growth. However, the extreme conditions of temperature (>80 °C) and NaOH concentration (50%) reported in the literature were not suitable for extraction of chitosan from *P. magdalenae* scales; therefore, it was necessary to develop a new method to obtain it because chitin extracted from these scales was β-type at room temperature and 2 wt.% NaOH conditions, as shown in this work. These mild conditions allowed chitosan to be obtained with desirable physical and chemical characteristics, such as lower molecular weight at 107.18 kDa than that of commercial chitosan at 151.11 kDa, which had effects on the thermal properties (lower *T*_P_, 148.82 °C) but allowed its expected effect as an antibacterial agent to be achieved, owing to its low molecular weight.

## Figures and Tables

**Table 1:** Quantification of components other than chitin in *P. magdalenae* scales.

Component	*P. magdalenae* Scales (mg/kg)	Chitin (mg/kg)
Phosphorous	109.34 × 10^3^ ± 8638.30	7.64 ± 0.61
Calcium	131.79 × 10^3^ ± 4.372	1.95 × 10^3^ ± 4.37
Protein	314.10 × 10^3^ ± 9081.99	75.65 × 10^3^ ± 1454.28

**Table 2:** Molecular weight and deacetylation degree of chitosan extracted from *P. magdalenae* scales.

Sample	Deacetylation Degree (%)	Molecular Weight (kDa)
Chi-2%	94.91 ± 1.35	107.18 ± 24.99
Chi-4%	100.06 ± 4.60	134.58 ± 24.39
Chi-6%	100.99 ± 0.00	240.3 ± 134.02
Chi-commercial (control)	72.00 ± 2.98	151.11 ± 4.47

**Table 3:** Parameters extracted from DSC analysis of commercial chitosan and chitosan obtained from *P. magdalenae* scales.

Sample	*T*_O_ (°C, Onset)	*T*_P_ (°C, Peak)	*T*_C_ (°C, Completion)	∆*H* (J/g Dry Weight, Enthalpy)
Chi-commercial	182.85	186.25	199.00	195.66
Chi-2%	144.82	148.42	170.51	143.23

## Data Availability

The data presented in this study are available on request from the corresponding author.
